# Hebbian Plasticity Realigns Grid Cell Activity with External Sensory Cues in Continuous Attractor Models

**DOI:** 10.3389/fncom.2016.00013

**Published:** 2016-02-17

**Authors:** Marcello Mulas, Nicolai Waniek, Jörg Conradt

**Affiliations:** Neuroscientific System Theory Group, Department of Electric and Computer Engineering, Technische Universität MünchenMunich, Germany

**Keywords:** grid cells, grid realignment, spatial information processing, continuous attractor network, sensory integration

## Abstract

After the discovery of grid cells, which are an essential component to understand how the mammalian brain encodes spatial information, three main classes of computational models were proposed in order to explain their working principles. Amongst them, the one based on continuous attractor networks (CAN), is promising in terms of biological plausibility and suitable for robotic applications. However, in its current formulation, it is unable to reproduce important electrophysiological findings and cannot be used to perform path integration for long periods of time. In fact, in absence of an appropriate resetting mechanism, the accumulation of errors over time due to the noise intrinsic in velocity estimation and neural computation prevents CAN models to reproduce stable spatial grid patterns. In this paper, we propose an extension of the CAN model using Hebbian plasticity to anchor grid cell activity to environmental landmarks. To validate our approach we used as input to the neural simulations both artificial data and real data recorded from a robotic setup. The additional neural mechanism can not only anchor grid patterns to external sensory cues but also recall grid patterns generated in previously explored environments. These results might be instrumental for next generation bio-inspired robotic navigation algorithms that take advantage of neural computation in order to cope with complex and dynamic environments.

## Introduction

Since the discovery of grid cells in the medial entorhinal cortex (MEC) in 2005 (Hafting et al. 2005), three main classes of computational models were proposed in order to explain the underlying neural mechanisms in this area of the brain. The oscillatory interference model is based on the interaction of periodical theta rhythms that interfere with each other (Burgess et al., 2007). The continuous attractor network (CAN) model relies on recurrent connectivity, which is able to generate periodical bumps of activity on a 2D neuronal sheet that shift depending on the rat velocity (Fuhs and Touretzky, 2006). A third class of models is based on self-organizing principles to generate grid-like activity (Gorchetchnikov and Grossberg, 2007; Kropff and Treves, 2008). All models are supported by experimental evidence, but at the same time fail to predict other specific characteristics. As a consequence, the choice of a specific model directly depends on which experimental evidence is considered the most important.

In this work we focus on CAN models because they perform path integration, as suggested by experimental evidence (McNaughton et al., 2006) and do not require the generation of precise velocity-controlled oscillators, whose biological plausibility is still a matter of debate (Yartsev et al., 2011). However, two important electrophysiological observations highlight the limits of these models. First, CAN models are currently unable to generate grids aligned with landmarks in the environment. Experiments with rats exploring an arena show that in different sessions the activity of a specific grid cell produces the same spatial grid pattern in terms of spacing, orientation, and phase (Hafting et al., 2005). CAN models can generate grids with a constant spacing but their orientations and phases depend on the initialization of the simulation and not on specific features of the environment. Second, CAN models perform path integration based on velocity signals. As a consequence, the accumulation of errors in the velocity estimates makes it impossible to reproduce a stable grid pattern in space over long periods of time in absence of a corrective mechanism (Burak and Fiete, 2009; Hardcastle et al., 2015).

According to experimental evidence, environmental landmarks might be instrumental to anchor grid cell activity in space (Derdikman et al., 2009). In principle, grid cell models that perform path integration can exploit sensory information to reset the accumulated error due to inaccurate velocity estimates (Biegler, 2000). A possibility is that plastic connections from sensory areas are continuously involved in this corrective action. However, how exactly grid cells can align themselves with external sensory cues is still an open question (Moser et al., 2014). Hebbian learning was already used to successfully prevent drifts of the activity bumps in a model of head direction cells based on a 1-dimensional CAN (Skaggs et al., 1995). In this model so called visual cells provided the necessary excitation to appropriately affect the network activity and push it toward the right configuration. More recently, Hardcastle et al. (2015) showed that it is possible to stabilize the activity of grid cells in a continuous attractor model by introducing border cells to provide corrective spatial information. However, in this model the strength of excitation is computed a priori depending on the amount of grid network activity corresponding to the borders of the arena. In this paper, we asked if it is possible to combine the two approaches by using Hebbian plasticity and location specific sensory information to stabilize the activity of grid cells in a CAN model.

In order to test this hypothesis we implemented a minimal neural model composed of a grid cell network and a sensory map connected by plastic excitatory connections. From an anatomical point of view, the sensory information encoded by the sensory map plausibly originates from the lateral entorhinal cortex (LEC) that directly projects on to the hippocampus. In turn, the hippocampus could possibly provide grid cells with the sensory information necessary for correcting their activity (Bonnevie et al., 2013). However, for the purposes of this work we chose not to model the complex and still not fully understood interactions between grid cells in MEC and place cells in the hippocampus. As in Skaggs et al. (1995) and Hardcastle et al. (2015), we took advantage of the dynamic properties of continuous attractors to push the activity bumps toward the right configuration. The correction is applied by excitatory connections projecting on to grid cells from sensory units that encode for the presence of visible landmarks. These connections are strengthened depending on the coactivation of grid cells and sensory units.

Our simulations show that a Hebbian plasticity-based correction mechanism is not only able to stabilize spatial grid patterns for periods of time comparable with the duration of experimental sessions, but also to associate the same grid pattern to a specific environment. In addition to increasing the biological plausibility of our model, the main advantage of using Hebbian plasticity instead of a predefined stimulation rule is that any location specific sensory information can be used to realign grid cell networks. Given its capability to generate stronger connections between neural areas that are more durably associated, Hebbian learning can automatically assess over time the stability of external sensory information that encode for environmental landmarks. This turns out to be a crucial feature that can potentially improve robotic spatial navigation in complex and dynamic environments. Because of the important implications that this neural mechanism can have on robotic navigation, we validated our model not only with simulations but also with a more realistic robotic scenario.

## Materials and methods

### Neural modeling

**Figure 1:**
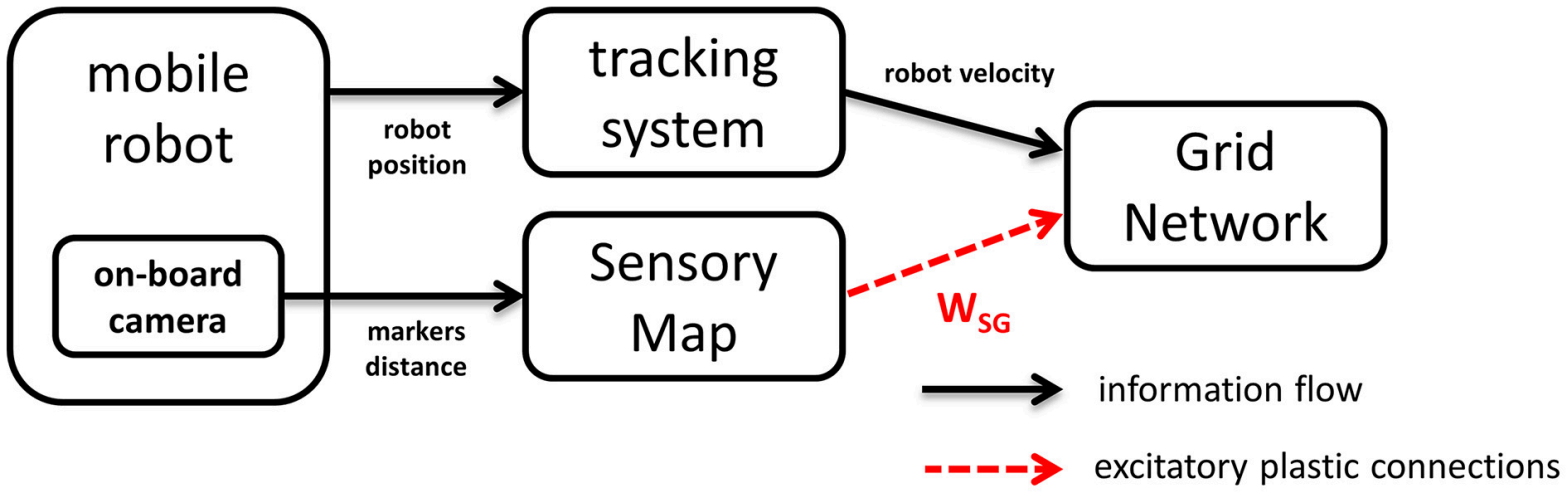
**Neural simulation modules**. The Grid Network receives robot velocity information from a tracking system. In addition it receives sensory information from the Sensory Map by means of excitatory plastic projections. The activation of the Sensory Map units depends on visual information provided by a camera on board the mobile robot.

Our neural model consists of two main components, a grid network and a sensory map interacting as shown in Figure 1. The grid network module is responsible for storing information about the position of the robot and receives information about its linear velocity and orientation. In order to evaluate the effectiveness of Hebbian plasticity to prevent the accumulation of path integration errors, we used both artificial and real data as input. In the real case scenario velocity information is estimated based on the robot trajectory as it is recorded by an external tracking system. In the simulated case it is computed based on the simulated trajectory of the robot. In both cases, the grid network receives excitatory plastic projections from the sensory map module, whose activation directly reflects the position of markers in the field of view of the camera (real or simulated). The sensory map is organized in a topographic way, thus preserving spatial correlations in the sensory stream.

The grid cell network module stores and computes spatial information by performing path integration on the robot velocity. Its implementation is based on the CAN model described by Burak and Fiete (2009) using leaky integrate and fire neurons instead of Poisson neurons. The model consists of four 2-dimensional networks of 64 × 64 neurons. Equation (1) shows the differential equation we used at every iteration (iteration step of 1 ms) to update the membrane potential *u*_*m*_ for all grid cells.

(1) $$\begin{array}{r} \tau_{m}\frac{du_{m}}{dt}=-u_{m}\left(t\right)+u_{rest}+R_{m}I\left(t\right) \end{array}$$

Whenever *u*_*m*_ exceeds the threshold potential *u*_*th*_ an action potential is fired and propagated to all postsynaptic neurons with a delay ranging from 1 to 5 ms. In addition, we set *u*_*m*_ equal to *u*_*reset*_ for the whole duration of the refractory period τ_*ref*_. Table 1 lists the values of the biophysical parameters we used in our simulations.

**Table 1:** **List of neural model parameters**.

**Parameter name**	**Value**
Membrane time constant (τ_*m*_)	10 ms
Membrane resistance (*R*_*m*_)	10 Ω
Resting membrane potential (*u*_*rest*_)	−65 mV
Threshold potential (*u*_*th*_)	−63 mV
Reset potential (*u*_*reset*_)	−67 mV
Refractory period (τ_*ref*_)	5 ms
Baseline current (*I*_*b*_)	2 mA

As Equation (2) illustrates, the current *I* in input to each neuron is the sum of the baseline current *I*_*b*_, the sensory current *I*_*s*_, and the robot velocity-dependent current *I*_*v*_.

(2) $$\begin{array}{r} I\left(t\right)=I_{b}+I_{s}\left(t\right)+I_{v}\left(t\right) \end{array}$$

The baseline current *I*_*b*_ is an unspecific global constant excitation that is necessary to elicit network spontaneous activity even in absence of any other excitatory input (Bonnevie et al., 2013). The sensory current *I*_*s*_, computed according to Equation (7), depends on the activation of the sensory map and on the plastic connectivity between grid cells and sensory units. In addition, all neurons of each of the four 64 × 64 networks of the model receive an excitatory input current *I*_*v*_ that is proportional to the robot velocity component in one of the four possible directions in a 2D space (i.e., N, W, S, and E). Equation (3) shows how we computed *I*_*v*_ as a function of the robot forward velocity *v* and the robot orientation ϑ.

(3) $$\begin{array}{r} I_{v}\left(t\right)=\gamma \;v\;\left(t\right)\;\cos\;\left(\vartheta \left(t\right)-\theta \right) \end{array}$$

We tuned the velocity-dependent current gain γ in order to balance the excitation levels in the grid network. θ is a network constant parameter that can assume one of four values (0°, 90°, 180°, 270°), corresponding to the four spatial directions.

The CAN model described by Burak and Fiete (2009) is based on recurrent connectivity to work. Each neuron inhibits all neighboring neurons inside a circular area. The center of the inhibition circle is shifted in the same direction θ as the robot velocity that modulates the neuronal input current. At the beginning of each simulation session neuronal state variables and parameters (e.g., membrane potentials *u*_*m*_ and transmission delays) are initialized with random values sampled from physiological ranges.

**Figure 2:**
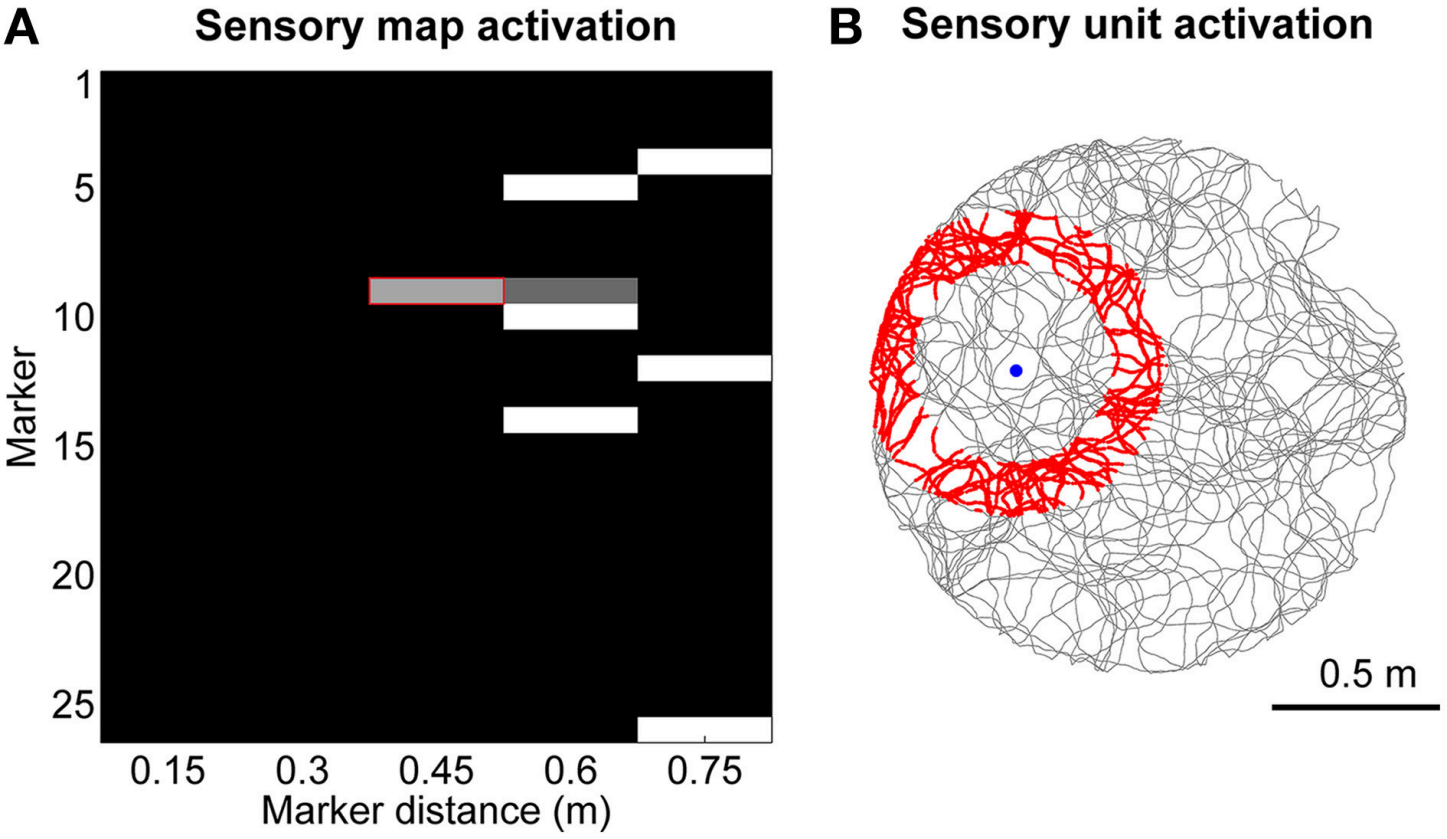
**Encoding of sensory information. (A)** Example of sensory map activation when the robot is moving toward marker #9. **(B)** Spatial activation (in red) for the sensory unit that encodes the distance between the robot and marker #9 approximately equal to 0.45 m.

The sensory map module consists of a 2-dimensional matrix of units that can assume a continuous value of activation between 0 and 1. It encodes location-specific sensory information with unique patterns of activation as shown, as an example, in Figure 2A. Each sensory unit *s*_*m, d*_ encodes for the presence of a specific digital marker *m* on the ceiling at a specific distance *d* from the visual field center of the robot camera, which is pointing upwards with a field of view radius *r*_*fov*_ equal to 0.75 m. Equation (4) shows the rule we defined to update the activation of sensory unit *s*_*m, d*_, where *d*_*m*_ is the distance between the robot and the marker *m*, and *n* (= 5) is the number of bins we chose to discretize the field of view. The two time constants τ_*active*_ and τ_*inactive*_ both equal to 50 ms control the activation and inactivation rates. In practice, when the robot is in the area of activation of a sensory unit (shown in red in Figure 2B as an example) the activation of the sensory unit will asymptotically converge to 1, whereas it will decay to 0 otherwise.

(4) $$\begin{array}{r} s_{m,d}=\{\begin{array}{c} \left(1-\frac{1}{\tau_{active}}\right)s_{m,d}+\frac{1}{\tau_{active}},\;\left|d_{m}-d\right|\leq \frac{r_{fov}}{2n}\\ \;\left(1-\frac{1}{\tau_{inactive}}\right)s_{m,d},\;\left|d_{m}-d\right|>\frac{r_{fov}}{2n} \end{array} \end{array}$$

We used 26 different digital markers and distances were discretized using five bins in order to test our stabilization mechanism in presence of coarse location specific information. This resulted in a localization accuracy of 48 pixels on the camera image and correspondingly to 13.2 cm on the ceiling where the markers were attached. Given that the sensory map encodes distances from the center of the camera's field of view, its activation pattern is theoretically invariant to rotations of the camera itself. However, in a real robotic setup slight inclination of the camera on the robotic frame resulted in slightly different sensory activation patterns for the same location depending on the orientation of the robot.

The connectivity between the grid network and the sensory map is based on a rate-based Hebbian plasticity rule, which is applied at every iteration of the simulation (time bin △*t* equal to 1 ms). Using Equation (5) we first compute the coactivation α_*ij*_ between the sensory unit *i*, with activation *s*_*i*_ (0 ≤ *s*_*i*_ ≤ 1), and the grid cell *j*, with instantaneous firing rate *r*_*j*_. The variable *r*_*max*_ is the maximum instantaneous firing rate recorded in the grid cell network for the current iteration and α_*th*_ is a coactivation threshold parameter set to 0.05.

(5) $$\begin{array}{r} \alpha_{ij}=s_{i}\cdot \frac{r_{j}}{r_{max}}-\alpha_{th} \end{array}$$

We then compute the change rate of the synaptic weight *w*_*ij*_ for the projection between sensory unit *i* and grid cell *j* using Equation (6), where we set the time constant τ to 10 s and we update the synaptic weight only if the coactivation α_*ij*_ is positive.

(6) $$\begin{array}{r} \frac{dw_{ij}}{dt}=\{\begin{array}{cc} 0, & \alpha_{ij}\leq 0\\ \frac{\alpha_{ij}\;-\;w_{ij}}{\tau }, & \alpha_{ij}>0 \end{array} \end{array}$$

This formulation of Hebbian plasticity makes the synaptic weights of the sensory projections asymptotically converge to the average coactivation between the corresponding sensory map unit *i* and grid cell *j*. We used Equation (7) to compute the amount of excitatory sensory current *I*_*s*_ injected in grid cell *j* at every iteration. *k* is a constant parameter (sensory current gain) equal to 0.05 that we tuned to balance sensory currents with velocity-dependent currents.

(7) $$\begin{array}{r} I_{s}=k\underset{i}{\sum }w_{ij}\cdot \alpha_{ij} \end{array}$$

In order to set critical parameters of the neural model (listed in Table 2), which, to the best of our knowledge, do not have a clear biological plausible value, we performed a brute-force parameter search running in parallel multiple simulations. We first set grid cell network parameters (e.g., recurrent connectivity radius and recurrent connection synaptic weights) to generate spatial grids with the highest gridness score for short simulated times. Next, we set Hebbian plasticity parameters (e.g., plasticity time constant τ and coactivation threshold α_*th*_) to have the best stabilization performance over longer simulation times. We found that some parameters are particularly critical for the proper working of Hebbian plasticity, as, for example, the plasticity time constant τ of Equation (6). On the one hand, too short time constants make the corrective mechanism interfere with the path integration of the grid network by preventing the continuous attractor to shift depending on the velocity-dependent currents. On the other hand, too long time constants make the stabilization mechanism too weak to prevent the accumulation of path integration errors.

**Table 2:** **List of model parameters tuned with a brute-force search approach**.

**Parameter name**	**Value**	**Description**
Recurrent connectivity radius	8	Radius of the inhibitory recurrent connectivity for each grid cell in the CAN
Recurrent connectivity shift	2	Shift in each of the four spatial directions of the recurrent connectivity of the CAN
Recurrent synaptic weight	−0.2	Synaptic weight of all recurrent connections between grid cells
Grid network baseline current (*I*_*b*_)	2.0 mA	Excitatory current injected at every iteration in each grid cell
Velocity-dependent current gain (γ)	0.02	Gain that controls the amount of velocity-dependent currents injected in each grid cell
Plasticity time constant (τ)	10000 s	Time constant that controls the weight change rate of a sensory synapse
Coactivation threshold (α_*th*_)	0.05	Minimum level of coactivation necessary to induce a plastic change in a sensory synapse
Sensory current gain (*k*)	0.05	Gain that controls the amount of sensory currents injected in each grid cell
Maximum sensory synaptic weight	0.5	Maximum allowable weight for a sensory synapse

The connectivity matrix between sensory map and grid cell network was initialized either to zero if the robot explored an unfamiliar environment or to a previously learned connectivity matrix if the robot explored a familiar environment. In this work we do not take into account the exploration of the robot of multiple environments.

### Robotic experiment simulation

**Figure 3:**
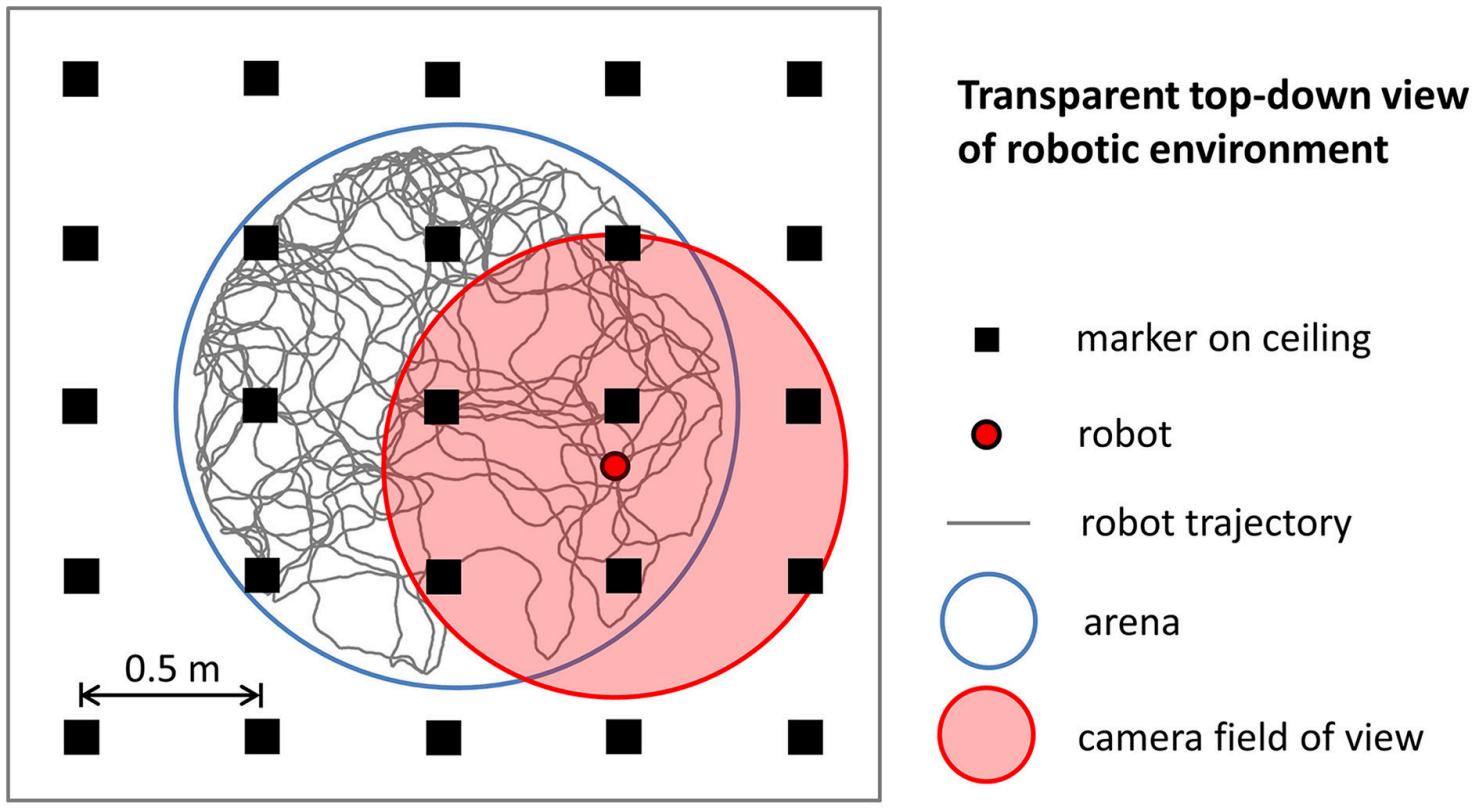
**Simulation of robotic experiments**. The trajectory of the robot is randomly generated within the boundaries of a circular arena. A virtual camera on top of the robot records the relative position within its field of view of virtual markers arranged in a rectangular grid.

**Figure 4:**
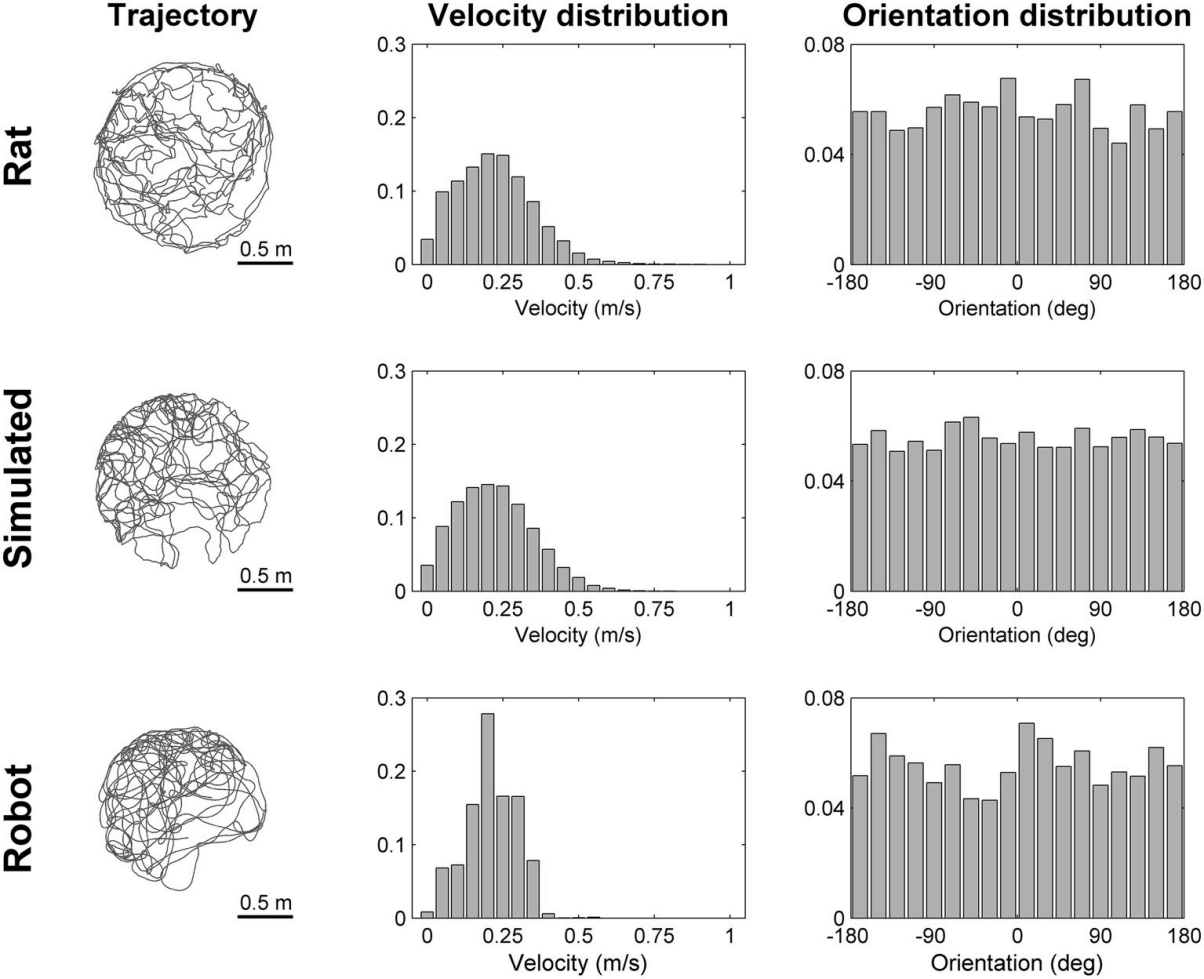
**Comparison of trajectories**. The trajectory, the distribution of velocities and the distribution of orientations are shown for a rat (**top row**), for a simulated robot (**middle row**), and for a real robot (**bottom row**) while exploring for 300 s a circular arena of the same size (diameter equal to 1.6 m). The simulated and real robot trajectories resemble the trajectory of a rat with different degrees of approximation (Experimental rat data adapted from Hafting et al., 2005).

To simulate robotic data we modeled the most important aspects of the experiments we performed using a real mobile robot. We intentionally did not model any source of noise that could make the simulation more realistic because the simulated data were intended to be used to validate our stabilization mechanism in ideal conditions. Figure 3 illustrates the main elements of the robotic experiment included in our simulation. Virtual markers, which cover the ceiling of the room containing the circular arena, are arranged in a rectangular grid and are equidistant (contiguous markers are 0.5 m apart). A virtual camera placed on top of the robot records their positions with a visual field equal to 1.25 m. The trajectory of the robot, modeled as a mobile point, is randomly generated within the boundaries of a circular arena (1.6 m diameter). We simulated the random trajectory of the robot so that it resembles the trajectory of a rat exploring a circular arena of the same size. Figure 4 shows that the two trajectories are similar in terms of distribution of velocity (top row: average rat velocity = 0.22 ± 0.13 m/s (mean ± s.d.), middle row: average simulated robot velocity = 0.22 ± 0.13 m/s) and the directions of movement are uniformly distributed.

### Robotic data acquisition

#### Robotic setup

**Figure 5:**
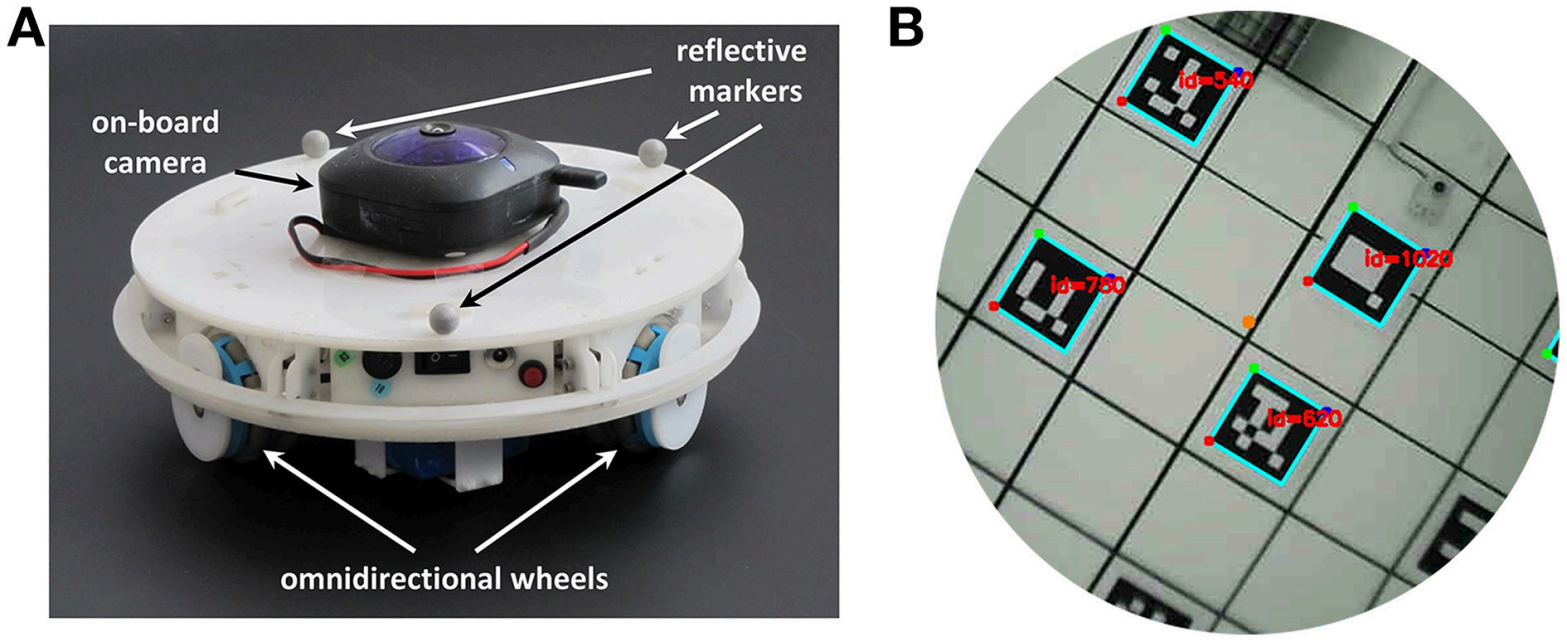
**Components of the robotic setup. (A)** Mobile robotic platform used to record sensory data during the exploration of a circular arena. A wireless camera pointing to the ceiling is mounted on top of the robot. Three reflective markers fixed on the robot frame are used by the tracking system to estimate the velocity and orientation of the robot. **(B)** The field of view of the camera mounted on top of the robot. The center of the camera's field of view is aligned with the center of the robot. The visible markers, arranged on a regular grid on the ceiling, are detected and decoded by the software *Aruco* (Garrido-Jurado et al., 2014).

To perform the experiments we used a mobile robot (240 mm diameter) that moves in a circular empty arena (1.6 m diameter). The robot, shown in Figure 5A, is equipped with three omnidirectional wheels located at the vertices of an equilateral triangle and has 360° range of movement. However, we limited its movements to make its trajectory more comparable to the one followed by rats while exploring a similar environment. More precisely, we controlled only its linear velocity in the forward direction and its angular velocity. The communication with the robot to send motor commands is made via a wireless connection.

In order to track the trajectory of the robot we used a 3D tracking system^1^ consisting of eight overhead cameras. The position and orientation of the robot can be estimated from the positions of three reflective markers (10 mm diameter) placed on top of its upper cover (Figure 5A). The tracking system can localize the robot with an accuracy of ~10 mm at a sampling frequency of 120 Hz.

An on-board wireless camera acquires visual information with a resolution of 640 × 480 pixels at a frame rate of 17.5 Hz (Figure 5B). The center of the camera's field of view is aligned with the robot center and the camera points in direction of the ceiling, which is covered by markers. The square markers have a dimension of 0.16 m and form a regular grid with an inter-node distance of 0.5 m. Each marker is a 5 × 5 binary matrix and encodes 10 bits of information. The remaining 15 bits are used for code correction and make it possible to recognize each marker in a reliable way. We used the software *Aruco* (Garrido-Jurado et al., 2014) to decode the identifier associated with each marker.

#### Data recording software

We used two distinct programs to perform the robotic experiments. The first one allowed us to control the robot, to record its trajectory and to acquire its sensory information. The second program executed offline neural simulations using the recorded data as input. The programs were written in C++ and executed on a Ubuntu Linux platform. To record trajectory and sensory data, we used an Intel Core i5-2500 quad-core processor at 3.3 GHz with 16 GB of RAM. For the neural simulations we used a computer equipped with 4 AMD Opteron 6380 processors (64 cores in total) at 2.5 GHz and 128 GB of RAM.

The data recorder program constantly controls both the linear and angular velocity of the robot in order to follow a predefined randomly generated trajectory that uniformly covers the whole area of the circular arena. In order for the robot to accurately follow the predefined trajectory we limited its speed at ~50 mm/s. The recorded speed data of the robot was multiplied by a factor of 4 and was used as input of the neural simulations. In this way, the average speed of the robot is comparable with the average speed of a rat exploring a similar environment as shown in Figure 4 [top row: average rat velocity = 0.22 ± 0.13 m/s, bottom row: average robot velocity (multiplied by a factor of 4) = 0.21 ± 0.08 m/s]. However, the distributions of velocities differ due to different dynamic characteristics of the robot (e.g., inertia, angular momentum), that make it difficult to precisely reproduce the kinematics of a rat.

In parallel with the control of the robot the on-board camera on top of the robot acquires pictures of the ceiling. The images are processed online to extract information about the positions of the markers. The positions of the markers expressed as 2D image coordinates and the position and orientation of the robot as it is estimated by the tracking system are stored to be used as input for offline neural simulations.

## Results

### Grid stabilization in unfamiliar environments

In order to validate our Hebbian plasticity-based realignment mechanism we first tested our extended CAN model with input data generated by a simulated robot that could move freely in a circular arena for 30 min. In one control condition the grid network performed path integration using only robot velocity information as input. In the other condition, the network activity not only performed path integration but it was also stabilized by sensory information recorded by a virtual camera on top of the robot. The distance of the markers from the robot was used to activate the corresponding units of the sensory map.

**Figure 6:**
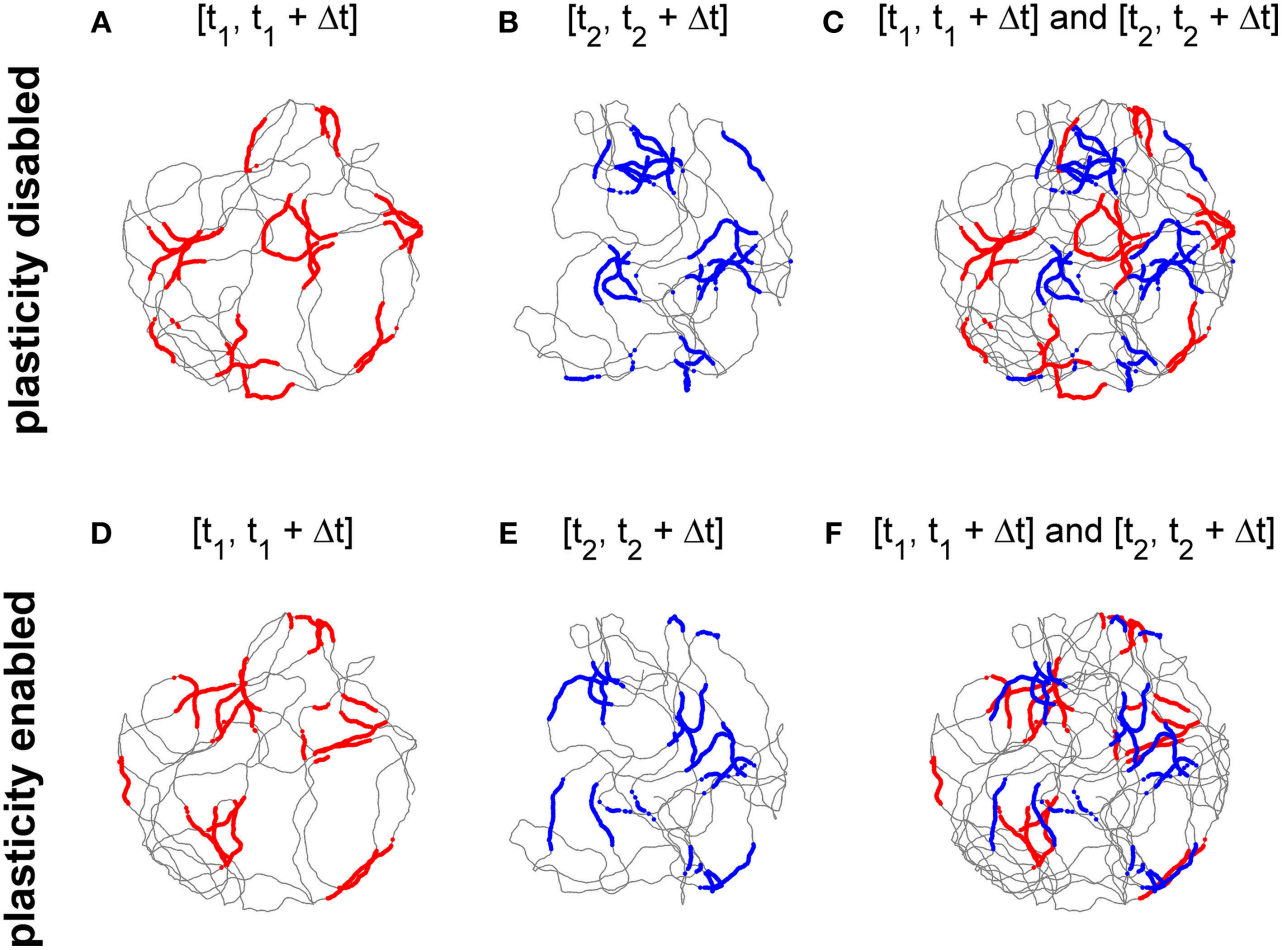
**The activation of the stabilization mechanism prevents the drift of spatial grid patterns. (A)** Spiking activity of a simulated grid cell without stabilization mechanism while a simulated robot explores a circular arena for 120 s (spiking activity in red, simulated trajectory in gray). **(B)** Spiking activity of the same cell of panel **(A)** after 10 min (spiking activity in blue). **(C)** The activities shown in panels **(A,B)** Do not overlap due to the accumulation of path integration errors. **(D)** Spiking activity of a simulated grid cell while a simulated robot explores a circular arena for 120 s with the activation of the stabilization mechanism. **(E)** Spiking activity of the same cell of panel **(D)** after 10 min. **(F)** The activities shown in panels **(D,E)** Overlap due to the Hebbian plasticity-based stabilization mechanism.

Top panels of Figure 6 show the activity of the cell at the center of the grid network when the stabilization mechanism was disabled. Figures 6A,B show grid-like patterns generated during short periods of exploration time (120 s) at two distinct times of the experimental session 10 min apart. The two grids have comparable gridness scores (0.725 and 0.736 respectively, computed as in Sargolini et al., 2006, where positive values indicate grid-like patterns). However, as shown in Figure 6C, the two grid patterns do not overlap due to the accumulation of path integration errors. Conversely, when the stabilization mechanism is enabled there is far greater overlap between the two grids of Figures 6D,E (gridness scores: 0.597 and 0.635 respectively) as shown in Figure 6F because the stabilization mechanism continuously corrects for the accumulation of errors in the path integration.

**Figure 7:**
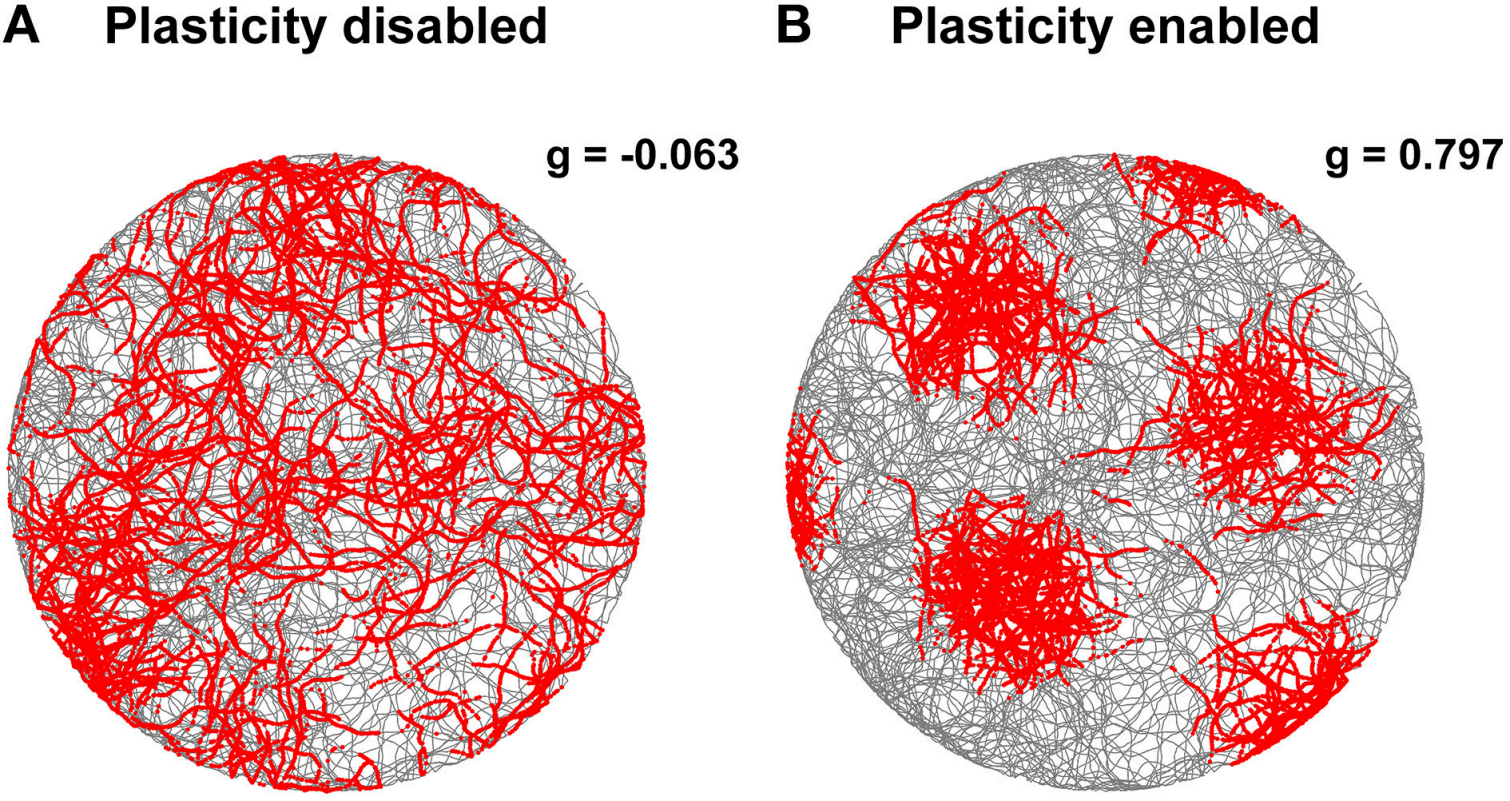
**Comparison of grid cell activity with and without stabilization mechanism for grid cell simulations with simulated data in input**. The spiking activities (red dots) of representative simulated grid cells are shown in correspondence to the position of a virtual robot (gray trace) while exploring for 30 min a circular arena without **(A)** and with **(B)** realignment mechanism (gridness scores: -0.063 for **A**, 0.797 for **B**). The activation of this plasticity-based stabilization mechanism successfully anchors the neural activity of grid cells to external sensory cues. This results in a well-defined grid pattern in space over long periods of neural simulation.

The stabilization effect can be observed even more clearly in Figure 7. This shows two representative examples of spatial grid patterns obtained in both conditions considering 30 min of spiking activity of a cell located at the center of the grid network. As shown in Figure 7A, without the activation of the realignment mechanism a hexagonal grid pattern is not visible anymore due to the drift of the grid activity. However, with the activation of the realignment mechanism the cell fires only in localized regions of the arena in line with the behavior of biological grid cells.

**Figure 8:**
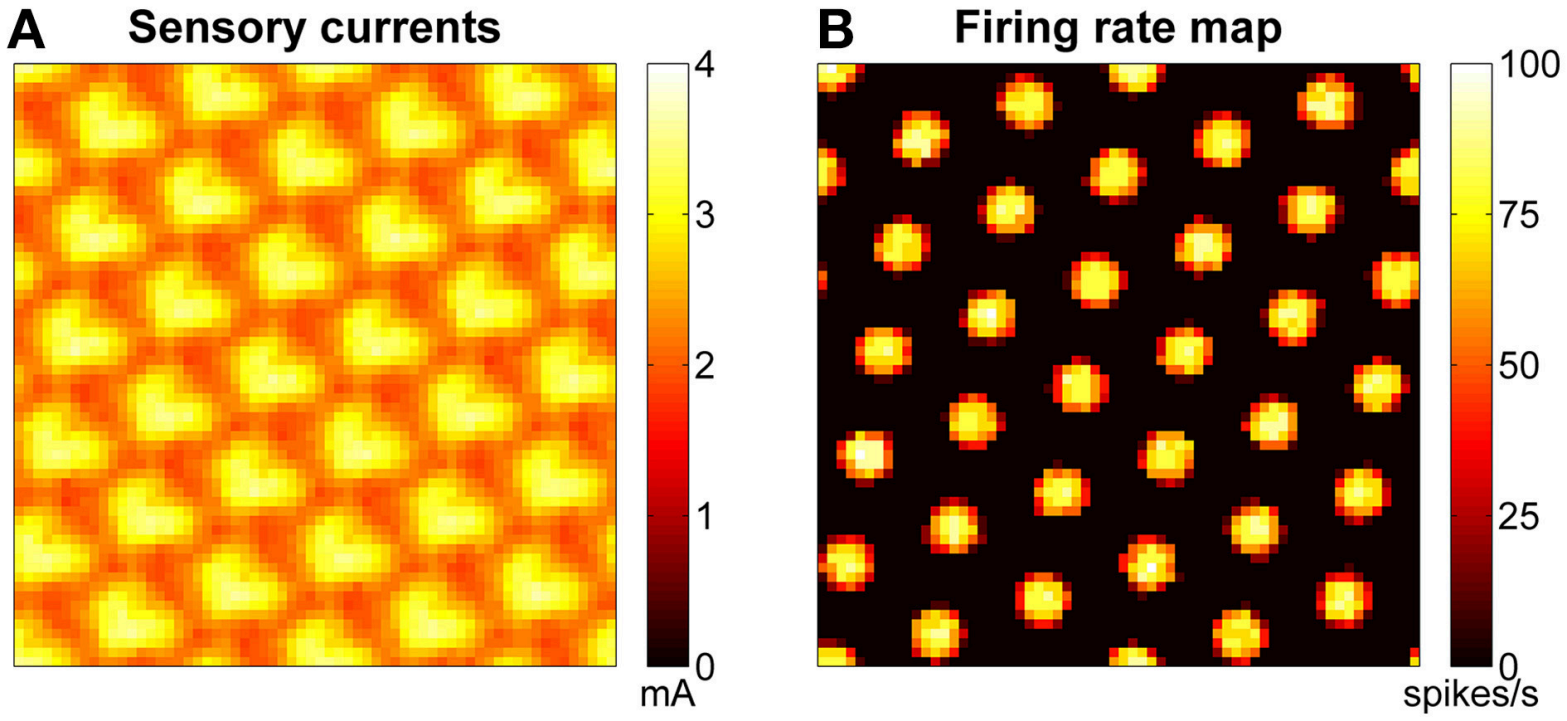
**Excitatory sensory currents push the activity of grid network toward the right configuration. (A)** Example of excitatory currents due to learned projections from the sensory map units to the grid cells after 30 min of robotic exploration of the arena: the bumps of excitation are arranged in a grid similar to that of the grid network activity (shown in **B**) but are not so well-defined. **(B)** Example of firing rate map for a network of grid cells. The spacing of the bumps in the grid network activity is related but not equal to the spacing of the grid pattern generated in space.

The stabilization mechanism is based on excitatory currents that depend at any given time on both the learned connections (between the grid network and the sensory map) and the current configuration of the sensory map. Figure 8A shows an example of excitatory currents due to such a learned connectivity after 30 min of exploration of the arena. The modulation of the excitatory sensory currents are closely related in terms of spacing and orientation to the firing rate map of the network, shown in Figure 8B.

**Figure 9:**
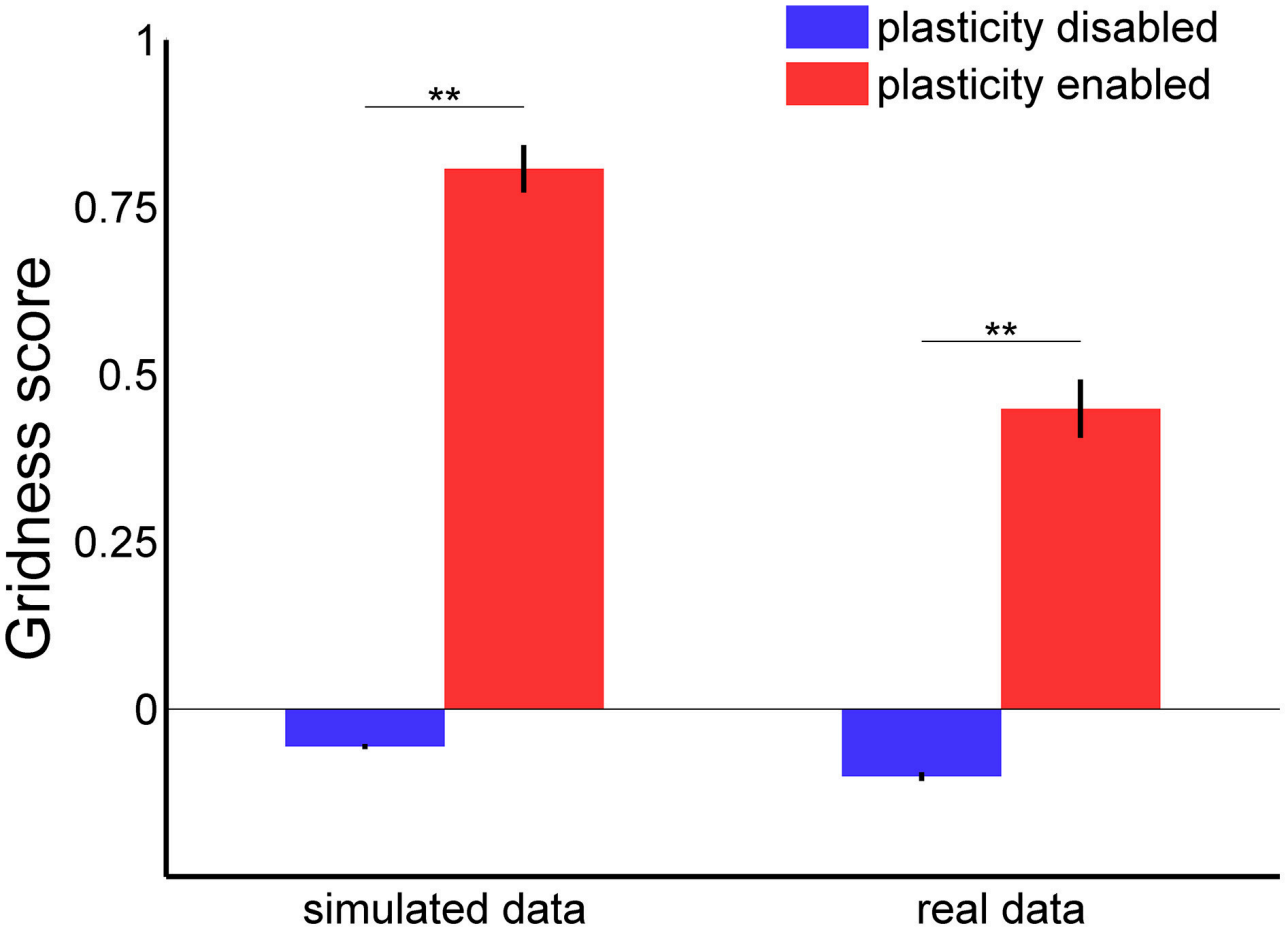
**Gridness score average with and without stabilization mechanism for grid cell simulations with simulated (left) and real data (right) in input**. For each experimental session we considered the activity of one single grid cell in the middle of the network. In both simulated and real conditions there is a significant difference in the average gridness score [one-way ANOVA, *F*_(3, 396)_ = 234.8 with Bonferroni correction, ^**^p < 0.01].

In order to confirm the stabilization effect of the learned connections, we analyzed the gridness score of spatial grid patterns generated by 100 grid cells during 30 min of simulated time with both the Hebbian plasticity mechanism enabled and disabled. The left side of Figure 9 shows a comparison of the average gridness score for both conditions with simulated data as input. A test of significance (ANOVA with Bonferroni correction, *p* < 0.01) confirmed the efficacy of Hebbian plasticity as a stabilization mechanism. On average, when the stabilization mechanism was disabled, the gridness score was equal to −0.056 ± 0.004 (mean ± s.e.m.) and the spatial pattern was similar to the one shown in Figure 7A. Conversely, when the stabilization mechanism was enabled, the gridness score was equal to 0.808 ± 0.036 and the grid pattern was similar to that given in Figure 7B.

**Figure 10:**
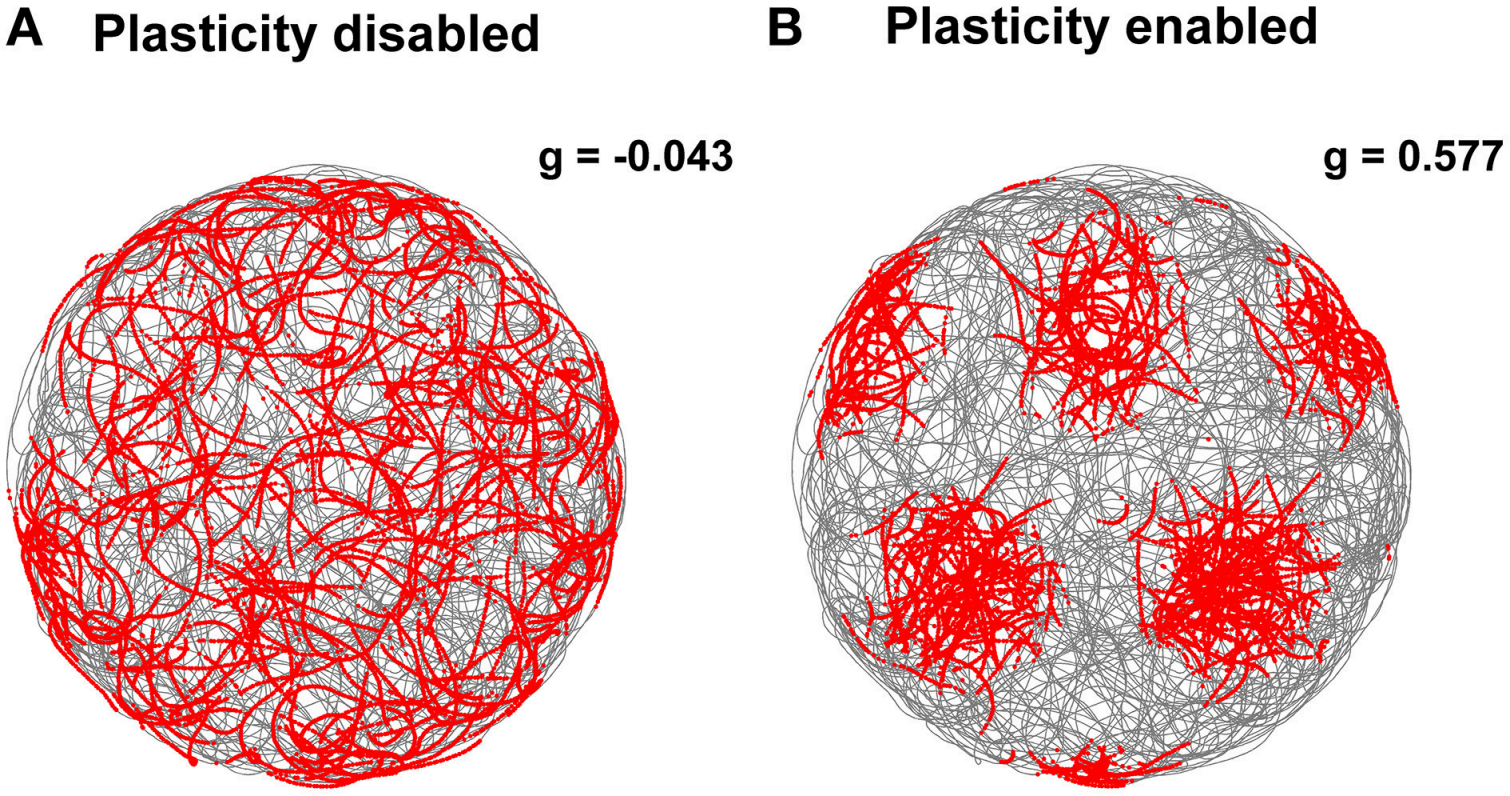
**Comparison of grid cell activity with and without stabilization mechanism for grid cell simulations with real data in input**. The spiking activities (red dots) of representative simulated grid cells are shown in correspondence to the position of a real robot (gray trace) while exploring for 30 min a circular arena without **(A)** and with **(B)** realignment mechanism (gridness scores: −0.043 for **A**, 0.577 for **B**). The activation of this plasticity-based stabilization mechanism successfully anchors the neural activity of grid cells to external sensory cues despite the additional noise introduced by imprecise velocity and landmark position estimates.

In addition, we tested our stabilization mechanism using real sensory data as input to the grid cell network simulation. More precisely, we estimated the robot velocity and orientation based on data recorded from a tracking system and the position of landmarks by processing the camera video stream. Notwithstanding the inaccuracy of the input data, the Hebbian plasticity mechanism was still able to stabilize the grid cell network activity. Figure 10 shows two representative examples of spatial grid patterns obtained when the stabilization mechanism was disabled (Figure 10A) and when it was enabled (Figure 10B). As previously shown for the simulated case (Figure 7), even in the presence of noisy inputs Hebbian plasticity was able to prevent the spatial grid pattern from drifting. We also analyzed the gridness score of spatial grid patterns generated by 100 simulated grid cells with real data in input. The right side of Figure 9 shows a comparison of the average gridness score between neural simulations with and without the stabilization mechanism. Similarly to the simulated case, there is a significant difference between the gridness of the two conditions (−0.101±0.007 for plasticity disabled, 0.449 ± 0.043 for plasticity enabled). The smaller gridness score average obtained with real instead of simulated data in input reflects the inaccurate sensory information provided by the robotic setup sensors.

**Figure 11:**
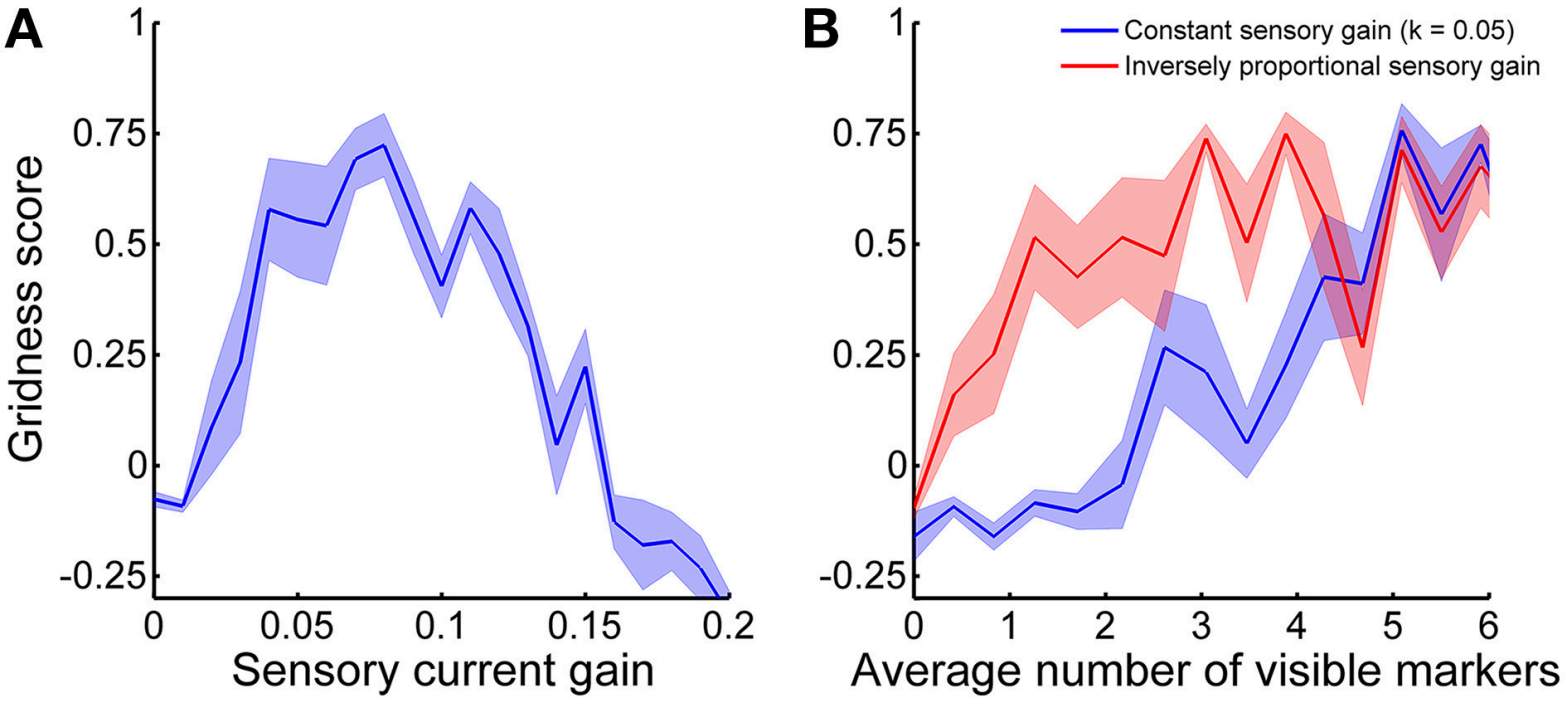
**Gridness score average as a function of (A) sensory current gain and (B) average number of visible markers**. The shadow areas represent the standard error of the mean. **(A)** The stabilization mechanism works best if velocity-dependent currents and sensory currents are balanced. **(B)** The optimal current sensory gain *k* depends on the average number of visible markers. The stabilization performance gets worse when the current sensory gain is constant (blue line) than when it is inversely proportional to the average number of visible markers (red line).

Both the velocity-dependent currents *I*_*v*_ and the sensory currents *I*_*s*_ are necessary for the CAN model to generate stable spatial grids over time. In fact, we tuned the respective gains with a brute-force approach to balance them and maximize stabilization performance. In order to clarify how critical the parameter tuning is for the stabilization we analyzed the gridness score of grid cell activity as a function of sensory current gain *k*. Figure 11A shows that there is an optimal value of *k* that yields the best stabilization results. If the sensory currents are either too small or too large the stabilization mechanism performed worse or did not work at all (negative gridness score). As a consequence, a balance between velocity-dependent currents and sensory currents seems necessary for the proper formation of the sensory connectivity.

In addition to the balance of input currents, our stabilization mechanism critically depends on the availability of a sufficient amount of sensory information. In principle, a robot needs to estimate the distances of only three markers in order to localize itself in space without ambiguity. Nonetheless, to test our stabilization mechanism we provided an average of visible markers equal to 6.3 ± 1.0 (mean ± standard deviation). Figure 11B shows the stabilization performance as a function of the average number of visible markers. We first varied the aperture diameter of the on-board camera from 0 to 1.5 m, while keeping all other simulation parameters constant (blue line). In this case, good stabilization performance (i.e., gridness score >0.5) was achieved with approximately more than five visible markers on average. However, in order to compensate for the decrease of sensory currents due to a fewer number of visible markers we also analyzed the gridness score adjusting the sensory current gain *k* according to the formula:

(8) $$\begin{array}{r} k=\frac{0.32}{n} \end{array}$$

where *n* is the average number of visible markers (red line in Figure 11B). In this other case, the stabilization mechanism performs well even with only about 1.5 visible markers on average.

### Grid realignment in familiar environments

According to experimental evidence the spiking activity of a grid cell recorded during different sessions but in the same environment generates spatial grid patterns with similar features in terms of spacing, orientation and spatial phase (Hafting et al., 2005). In contrast, the neural simulations presented so far generated spatial patterns with similar spacing, but random orientations and phases. This is due to the fact that the very first stable configuration of the grid network activity depends on the initialization of the cell membrane potentials, which are randomly set at the beginning of the simulations. Given that Hebbian plasticity can stabilize grid patterns in space by affecting the overall activity of the grid networks, we asked if the excitation provided by the sensory connectivity learned in a previous exploration of the arena would be also effective to realign the grid spatial pattern in order to match the first generated one.

**Figure 12:**
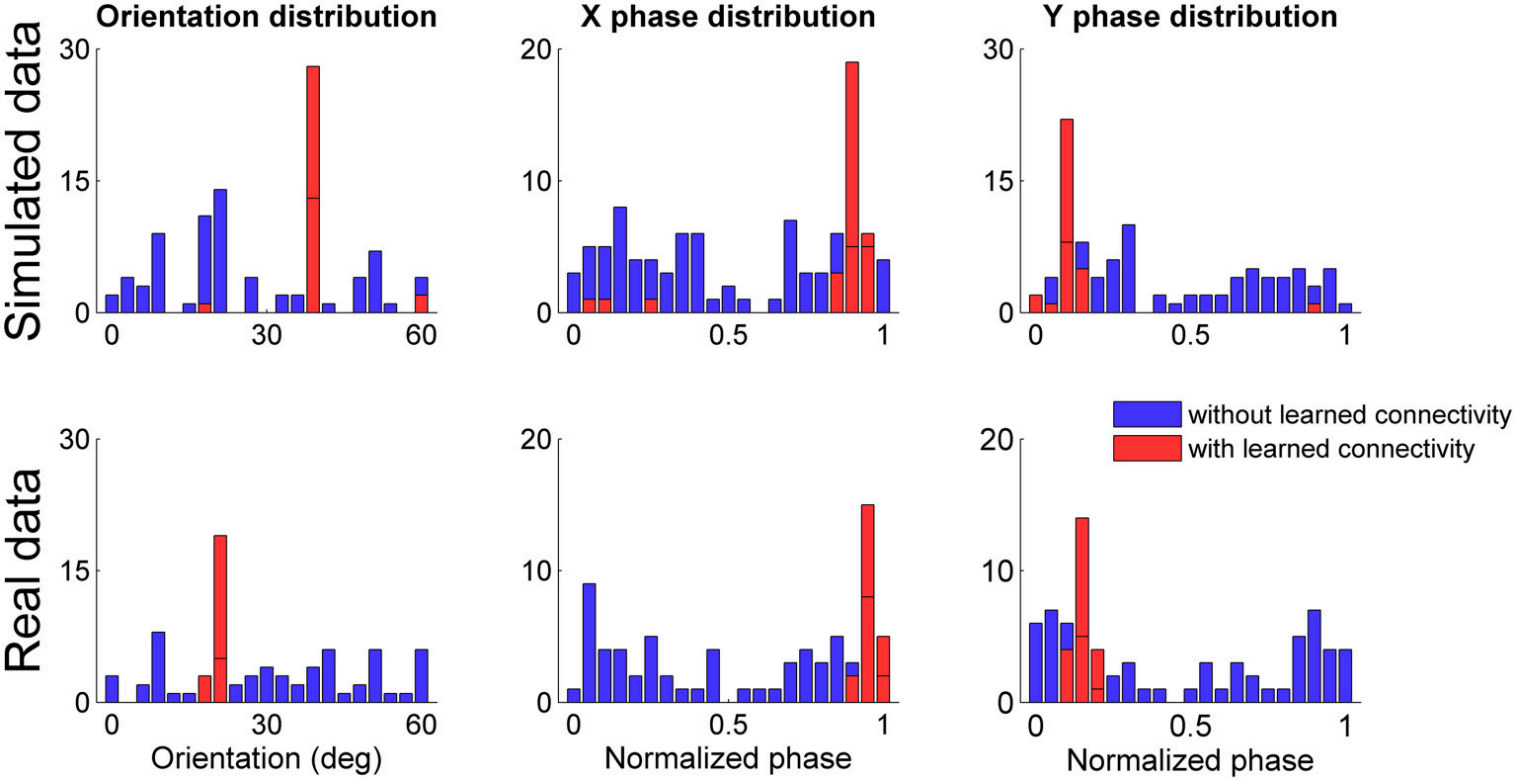
**Distribution of grid orientations and normalized spatial phases for spatial grids (with gridness score >0) generated by grid cells at the center of each simulated network**. Distributions refer to both simulated (**top panels**) and real data (**bottom panels**) in input, initializing the connectivity from the sensory map to the grid network to a zero matrix (in blue) and to a previously learned connectivity (in red). In both simulated and real conditions the previously learned connectivity drastically reduced the possible grid orientations and phases.

We simulated 100 grid networks for 30 min of simulated time after initializing the connectivity *W*_*SG*_ between the sensory map and the grid network with the connectivity learned in a previous simulation session that obtained a gridness score close to the average. As previously done for the tests in unfamiliar environments we considered neural simulations with both simulated and real data in input and for each simulation we analyzed only the spiking activity of the grid cell at the center of the network. Figure 12 shows the distributions of grid orientations and normalized spatial phases of grid patterns with positive gridness scores for both the case in which the connectivity between sensory map and grid network was initially set to a zero matrix (histograms in blue) and the case in which the connectivity was set to a previously learned connectivity (histograms in red). In both simulated and real conditions the previously learned connectivity drastically reduced the available orientations and phases and, as expected, the most frequent ones corresponded to those of the previously generated spatial grid. However, it is worth noting that the initialization of a simulation with a previously learned connectivity reduced the number of well-defined spatial grids. In both simulated and real data conditions the percentage drastically decreased (from 92 to 32% for the simulated data, from 76 to 22% for the real data). A plausible explanation is that the learned connectivity can correct the current grid network activity only if it does not already differ too much from the correct one.

## Discussion

Three main computational models based on different neural mechanisms are available to reproduce the most remarkable feature that distinguishes grid cells, i.e., the periodic hexagonal pattern defined in space by their spiking activity. As a consequence, in order to clarify the real biological mechanism behind grid cells, it is necessary to take into consideration further experimental evidence. One of the most important aspects to consider is how spatial grid patterns relate with the features of the surrounding environment. Electrophysiological experiments with rats exploring an arena showed that grid patterns are not only stable over time but also present non-random orientations and spatial phases. In fact, the same grid cell generates the same spatial grid pattern in different experimental sessions as long as the environment does not change. For this reason, neuroscientists hypothesized that the most salient sensory cues of the environment are used by grid networks in order to both stabilize and realign themselves. In this context Hebbian plasticity might be involved by establishing associations between grid configurations and environmental landmarks. However, how exactly this might work is still unclear.

In this work we considered a spiking model of grid cells based on continuous attractors, and we investigated how to stabilize and realign the network activity with location specific visual cues using Hebbian plasticity. Given that CAN models perform path integration based on estimates of the robot velocity that are affected by noise, they can reproduce spatial grids only for short periods of time because the accumulation of errors results in drift of the grid pattern. However, even with perfectly accurate, noise-free velocity input data the inherent stochastic computation of spiking neurons inevitably introduces errors in the processing of velocity information. Therefore, a stabilization mechanism is needed to periodically reset the accumulation of path integration errors. We implemented such stabilization mechanism by exploiting the capability of excitatory currents to affect the configuration of a CAN model of grid cells. The injected sensory currents push the current stable configuration of the CAN toward the correct one that is stored in plastic connections. This online learning mechanism relies on the relative stability of the spatial grids for sufficiently long periods of time, which is necessary for Hebbian plasticity to durably associate sensory information with grid cell activity.

We validated our Hebbian plasticity-based stabilization mechanism using simulated as well as real data. Our results show that Hebbian plasticity is effective at stabilizing the activity of grid cells even in the presence of noise. Excitatory plastic connections encoding sensory information about environmental landmarks continuously correct the activity of grid cells to prevent the drift of the spatial patterns. In this work we used a regular grid of visual markers to provide location specific sensory information to a robot. The regularity of the markers arrangement assures that there are always at least three markers visible to the robot at any given time. Indeed three markers are sufficient to resolve the robot positional ambiguity due to sensory information depending only on distances. However, the precise position of each marker does not have any influence on the spatial features of the hexagonal grid pattern generated by grid cells. In fact, we obtained very similar results for experiments with different marker distributions (e.g., with a sensory map encoding the distances of the corners of a square arena, or encoding both distance and direction of a single visual cue, data not shown).

In addition to stabilizing, the connectivity generated by Hebbian plasticity is also effective at realigning grid cell activity or, in other words, at recalling a previously generated spatial grid pattern in terms of spacing, orientation and spatial phase. However, in order for this stabilization mechanism to work, it is necessary to precisely define many biophysical parameters of our neural simulations. A careful parameter tuning is important for the grid cell model to reproduce spatial grid patterns even for short periods of time, regardless of the accumulation of path integration errors. In biological neural systems, critical biophysical parameters might have been fine-tuned over the course of evolution. Alternatively, a fine-tuning of parameters might be necessary in our simulations to provide for the lack of unknown homeostatic mechanisms that are not captured by the CAN model of grid cells.

Our validation tests with input data generated by a robotic setup helped us to better understand the limitations of using simulated data to investigate neural computation. Even if it is theoretically possible to accurately simulate real input data, it is difficult to predict a priori which deviations from ideality are important to include in the model to make the simulation more realistic. A substantial part of our work consisted in understanding how exactly real data differed from simulated one. For example, we found that the velocity of the robot should be greater than a certain threshold in order for the spiking CAN model to properly integrate velocity signals. As a consequence, electrophysiological experiments that systematically investigate the non-linear effect of low speeds on the gridness of spatial grid patterns might provide useful data to validate different models of grid cells. A second example occurred due to the specific geometry of our robotic experimental setup. An unnoticeable tilt of the on-board camera (about 4°) resulted in variations of marker localization estimates up to 0.4 m that made it more difficult for Hebbian plasticity to work as a stabilization mechanism. A more accurate set up of the camera on the mobile robot frame was necessary to fix the problem. Even if this problem was specific for our robotic setup, it stresses the importance of a more sophisticated preprocessing of sensory information adaptable to the configuration of the robot's sensors in order to provide more accurate estimates of the position of landmarks in the surrounding environment.

As our results show, Hebbian plasticity can be used to integrate robot velocity signals with sensory information in a neural system. Therefore, simultaneous localization and mapping algorithms can take advantage of neural computation to solve the loop closure problem, i.e., to recognize the same location after having traveled for a long path. In fact, the stability of a grid pattern in space is direct evidence that our stabilization mechanism makes a CAN model reliably store spatial information for longer periods of time. In principle, a robot can localize itself based only on sensory information. Nevertheless, in a real environment the position of landmarks is known with finite precision. As a consequence, combining two independent sources of information on the robot position (i.e., path integration and sensory-based position estimation) can not only increase the accuracy of the localization but also potentially make it more robust to sensory variability. However, as the results shown in Figure 11 suggest, the optimal combination of these sources of information requires a critical balance between velocity-dependent currents and sensory currents. In more realistic scenarios, when sensory information is not uniformly distributed as in our robotic setup, this balance might require adaptable current gains dynamically depending on the stream of sensory information in input.

In order for a robot to be autonomous, velocity information should be estimated based on on-board sensors instead of using an external tracking system. In this regard an accurate estimate of the orientation of the robot using biologically plausible methods could be possible by introducing an additional CAN model of head direction cells. As shown already by Skaggs et al. (1995) a similar Hebbian plasticity mechanism can stabilize and realign the activity of head direction networks as well.

We showed that Hebbian plasticity can account for experimental evidence that is not possible to get reproduced with a basic CAN model of grid cells, such as the stability of the spatial grid over time and its realignment in previously explored environments. By using more complex and dynamic environments we will assess the capability of Hebbian plasticity to account for other experimental observations such as the deformation of grid patterns in stretched environments (Barry et al., 2007) or the merging of two grid patterns after the removal of a separation between two contiguous environments (Carpenter et al., 2015). However, there are other electrophysiological observations that at the moment cannot be explained by our current extension of the model. As an example, possible orientations of a spatial grid seem to be distributed in discrete steps of 7.5° with respect to environmental landmarks (Stensola et al., 2012). Further refinements of the existing models are then required to fully clarify the neural mechanisms behind grid cells.

## Author contributions

MM designed the research work, implemented the simulations, performed the experiments and the analyses, and wrote the manuscript. NW designed the research work, built the robotic platform, and revised the manuscript. JC designed the research work, built the robotic platform, and revised the manuscript.

### Conflict of interest statement

The authors declare that the research was conducted in the absence of any commercial or financial relationships that could be construed as a potential conflict of interest.

## Footnotes

^1^“OptiTrak Tracking System,” website: http://www.optitrack.com
